# Gut Microbiota as a Key Modulator in the Pathophysiology of Sepsis: SURVEIL Project

**DOI:** 10.1002/mbo3.70301

**Published:** 2026-04-30

**Authors:** Chiara Bazzano, Alice Caramaschi, Nadia Massa, Silvio Collani, Marta Mellai, Nadia Barizzone, Andrea Rocchetti, Paolo Bottino, Maria Simona Caroppo, Valeria Bonato, Rosanna Vaschetto, Sara Scaglione, Alessia Francese, Narges Gholami, Claudio Caria, Paola Bianchi, Cristiano Lauritano, Luigi Castello, Annalisa Roveta, Antonio Maconi, Stefano Andreoni, Sandra Dalfonso, Elisa Bona

**Affiliations:** ^1^ Department for Sustainable Development and Ecological Transition University of Piemonte Orientale Vercelli Italy; ^2^ Department of Sciences and Technological Innovation University of Piemonte Orientale Alessandria Italy; ^3^ Center for Translational Research on Autoimmune and Allergic Diseases University of Piemonte Orientale Novara Italy; ^4^ Department of Health Sciences University of Piemonte Orientale Novara Italy; ^5^ Microbiology Unit University Hospital “Santi Antonio e Biagio e Cesare Arrigo” Alessandria Italy; ^6^ Microbiology Unit University Hospital “Maggiore della Carita” Novara Italy; ^7^ Simple Departmental Structure Research Laboratories—Integrated Activities Research and Innovation Department University Hospital “Santi Antonio e Biagio e Cesare Arrigo” Alessandria Italy; ^8^ Department of Translational Medicine University of Piemonte Orientale Novara Italy

**Keywords:** aging, colonization, dysbiosis, gut microbiota, multidrug‐resistant organisms, sepsis

## Abstract

Sepsis is a life‐threatening condition frequently associated with gut dysbiosis and bacterial colonization by multidrug‐resistant organisms. However, the interplay between gut microbiota, colonization patterns, and sepsis onset remains poorly defined. The authors analyzed longitudinal gut microbiota profiles from 132 hospitalized patients enrolled in the SURVEIL study. Rectal swabs were collected at three time points (baseline, sepsis onset, and discharge). Bacterial colonization status and MDR strains were determined through culture‐based methods, while microbiota composition was assessed *via* 16S rDNA sequencing. Diversity indices, taxonomic and functional profiles, and differential abundance analyses (LEfSe) were integrated with clinical metadata, including age and sepsis status. At baseline, colonized patients—particularly those harboring Gram‐positive taxa—exhibited significantly reduced alpha diversity compared to non‐colonized individuals. Aging further modulated diversity and beta diversity patterns independently. Sepsis was associated with profound dysbiosis, characterized by enrichment in opportunistic genera (e.g., *Finegoldia* sp., *Anaerococcus* sp., *Parabacteroides* sp.), reduced microbial diversity, and distinct beta diversity trajectories. Functional predictions revealed enhanced representation of anaerobic metabolism, nitrogen/sulfur cycling, and host‐adaptive traits in colonized states. MDR strains partially overlapped with bloodstream pathogens in septic patients, suggesting a possible link between intestinal colonization and bloodstream infection that warrants mechanistic validation. Our findings demonstrate that bacterial colonization and sepsis are strongly associated with compositional and functional shifts in the gut microbiota. Age and MDR carriage further shape microbiota dynamics. Early microbial signatures, such as *Finegoldia* sp. enrichment in colonized non‐septic patients, may represent early markers of microbial destabilization and sepsis risk.

## Graphical abstract

Longitudinal profiling of 132 hospitalized patients reveals that age, colonization status, and sepsis interact to shape gut microbiota diversity and composition. Sepsis is associated with reduced alpha diversity, beta‐diversity shifts, functional predictions (methanogenesis, nitrate/sulfate respiration), and partial MDR overlap with bloodstream pathogens, highlighting microbiota dynamics as potential risk biomarkers.

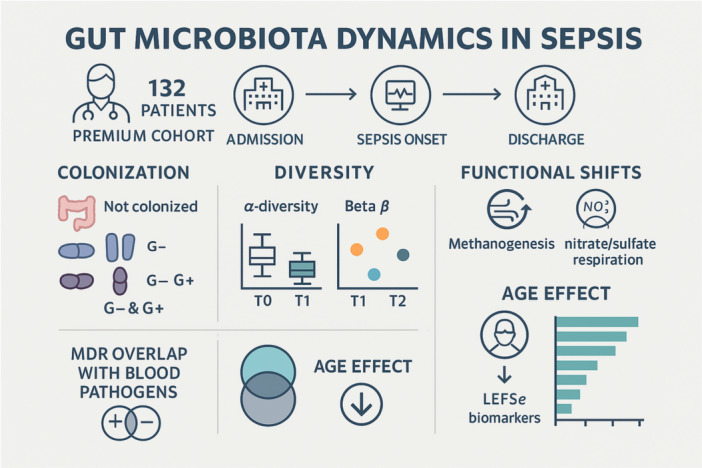

## 1 Introduction

Sepsis, a life‐threatening condition arising from a dysregulated host response to infection as defined by The Third International Consensus Definitions for Sepsis and Septic Shock (Sepsis‐3) (Singer et al. 2016), represents a critical challenge in modern medicine. The syndrome encompasses a wide spectrum of clinical presentations, ranging from mild organ dysfunction to full‐blown septic shock and multiorgan failure. Despite considerable advances in antimicrobial therapies, critical care practices, and early recognition protocols, sepsis continues to be a major cause of morbidity and mortality worldwide. It is recognized not only as a leading cause of death in hospitals but also as a major contributor to long‐term disability in survivors, who often suffer from persistent physical, cognitive, and psychological impairments, collectively known as post‐sepsis syndrome.

Globally, sepsis poses an immense public health burden. In a landmark analysis, Rudd et al. (2020) reported that in 2017, sepsis accounted for approximately 49 million cases and 11 million deaths worldwide—about 1 in 5 deaths globally. Although often associated with low‐ and middle‐income countries due to higher prevalence of infectious diseases and limited access to healthcare, sepsis also exerts a heavy toll in high‐income nations. Hospital‐acquired infections, aging populations, and rising antimicrobial resistance contribute to the persistently high incidence of sepsis in developed healthcare systems. In Europe, the situation is similarly concerning. Fleischmann‐Struzek et al. (2020) estimated more than 3 million cases and over 680,000 sepsis‐related deaths annually. However, these numbers likely underestimate the true burden, given limitations in diagnostics, inconsistent reporting, and inadequate surveillance systems.

In Italy, the incidence of sepsis has increased steadily over the last two decades (Istituto Superiore di Sanità 2023), paralleling demographic shifts such as population aging and the growing burden of chronic diseases. Moreover, Italy is considered a hotspot for antimicrobial resistance in Europe, further complicating sepsis management and increasing mortality risk. Regional disparities in healthcare infrastructure and implementation of sepsis protocols further affect outcomes.

Increasing attention has been directed toward the role of the gut microbiota in the development and progression of sepsis. The human gastrointestinal tract harbours a diverse and dynamic microbial community essential for immune homeostasis, metabolic balance, and intestinal barrier integrity (Belkaid and Hand 2014; Sommer and Bäckhed 2013). During critical illness, including sepsis, the gut microbiota undergoes profound alterations—a state referred to as dysbiosis (Haak and Wiersinga 2017; Zaborin et al. 2014). Dysbiosis involves a marked reduction in microbial diversity, loss of beneficial commensals, and overgrowth of opportunistic pathogens. Disruption of the gut barrier may result in microbial translocation and systemic spread of endotoxins and pathogens, amplifying inflammation and contributing to multiple organ dysfunction syndrome (MODS).

The gut has thus been proposed as both a source and target of injury in sepsis (Alverdy and Krezalek 2017). Several microbial taxa are thought to exert protective effects—such as *Faecalibacterium prausnitzii*, *Bifidobacterium* spp., *Lactobacillus* spp., and *Akkermansia muciniphila*—while others, including *Enterococcus* spp., *Clostridioides difficile*, *Escherichia coli*, *Klebsiella* spp., and *Staphylococcus aureus*, are linked to gut barrier damage and systemic inflammation (Shimizu et al. 2020a; Haak and Wiersinga 2017; Schuijt et al. 2016; Zaborin et al. 2014).

Preclinical studies show that modulating the gut microbiota—via probiotics, prebiotics, dietary intervention, or fecal microbiota transplantation—can attenuate systemic inflammation and improve outcomes in sepsis (Schuijt et al. 2016). However, clinical translation remains limited and demands mechanistic insight. Moreover, emerging research suggests that microbiota composition affects the pharmacodynamics of antibiotics and immunotherapies, underscoring the need for personalized approaches.

As we confront the global challenge of sepsis, integrating microbiome research into sepsis management represents a promising strategy. In this context, the aim of this study is to identify specific gut microbiota features predictive of sepsis development, including bacterial colonization by multidrug‐resistant strains.

## 2 Material and Methods

### 2.1 Standard Protocol Approvals, Study Participants and Samples Collection

This research is part of the SURVEIL project “SvilUppo di una piattafoRma di Vigilanza gEnomica per IL contrasto della pandemia covid‐19”, supported by Regione Piemonte “Azione 173, “*INFRA‐P realizzazione, rafforzamento e ampliamento Infrastrutture di ricerca pubbliche*”. The study was conducted as part of the SURVEIL project, coordinated by the University of Eastern Piedmont. The protocol was reviewed and approved by the Clinical Trial Center of the “Azienda Ospedaliera SS. Antonio e Biagio e Cesare Arrigo” of Alessandria (Italy). Ethical approval was granted under protocol number 0009474 of 29/04/2022, and all procedures were carried out in accordance with the Declaration of Helsinki and the principles of Good Clinical Practice (GCP).

The study was registered in the institutional Clinical Trial Center Registry (www.clinicaltrials.gov) under registration number NCT 07183722, in compliance with international standards for human research transparency and reproducibility. Written informed consent was obtained from all enrolled participants or their legal representatives prior to sample collection. Participants were recruited from June 2022 to August 2023 by the SURVEIL study group, established through the collaboration of two Italian hospitals (Santi Antonio e Biagio e Cesare Arrigo Hospital in Alessandria and Maggiore della Carità Hospital in Novara). The general cohort is composed of adult patients (18 years and above). Inclusion criteria: Age ≥ 18 years; patients admitted to the following Departments: General Anaesthesia and Reanimation Unit (RIA), Internal Medicine (IM), Cardio‐Thoracic‐Vascular Anaesthesia and Reanimation Unit (CCHI) of the Azienda Ospedaliera di Alessandria and Anaesthesia and Reanimation and Intensive Care Unit of the Ospedale Maggiore della Carità di Novara; signature of informed consent to participate in the study. Exclusion criteria: absence of signature of informed consent.

Samples were collected at baseline (T0): on admission of the patient to the wards of the participating centres, the study protocol was: (i) explained and written informed consent was signed by all patients meeting the inclusion criteria; (ii) a rectal swab was taken, which is part of normal clinical practice, for the study of colonisation by MDR bacteria; (iii) a rectal swab was taken specifically for the metagenomic analysis of the intestinal microbiota. Moreover, demographical information (i.e.,: date of birth, sex, age) were obtained for each participant. At the time of the diagnosis of bacteremia (T1) in any modality detected (Gram, Film array, direct Maldi TOF on blood, automatic T2dx system): patients who developed bacteremia during hospitalisation to the exclusion of those microorganisms that will be considered as contaminants from a microbiological point of view, a second rectal swab specifically for metagenomic analysis of the gut microbiota was performed. At time of discharge (T2) a final rectal swab for metagenomic analysis of the gut microbiota was performed on all colonised and non‐colonised patients who would not develop sepsis. Samples were stored at −80° till DNA extraction.

### 2.2 DNA Extraction and Next Generation Sequencing Analysis

DNA was extracted using QIAmp PowerFecal Pro DNA Kit by Qiagen (QIAGEN, GmbH, Hilden, Germany) and libraries for 16S rDNA sequencing were prepared according to the manufacturer's instructions using Microbiota Solution B kit, which amplifies V3‐V6 hypervariable regions of the 16S rDNA gene, provided by Arrow Diagnostics srl (Genoa, Italy). Sequencing has been performed on Illumina MiSeq platform (V2 Reagent 500 cycles—2 × 250 read length), using Phyx as internal standard.

### 2.3 16S rDNA Sequence and Statistical Analyses

Raw sequences were processed by MicrobAT v. 1.1.0 (Microbiota Analysis Tool), provided by SmartSeq Srl (SmartSeq Srl, Novara, Italy), that performed also primary analyses and quality controls. Sequences were aligned against Ribosomal Database Project (RDP v.11.5) database, if the following criteria were met query coverage ≥ 80% and similarity ≥ 97% (La Vecchia et al. 2025). Sequences with a read length < than 200 nucleotides or with a low quality (average Phred quality score < than 25) (Ewing et al. 1998) were removed. Sequences aligned to the reference database (Cole et al. 2014) were used to generate three files (OTU, taxonomy, metadata) that represent the input for the statistical analyses (Bona et al. 2021; Torre et al. 2022). Statistics were performed using R (v4.5.1) the R package “microeco” (Liu et al. 2021). Features having low count (< 30 counts), based on median value, and low variance measured using inter‐quantile range, were removed as low quality or uninformative features. Data were rarefied based on SRS (scaling with ranked subsampling) method (Beule and Karlovsky 2020).

The relative abundance at phylum and at genus level was calculated and the Kruskal–Wallis test was employed to assess the statistical significance among different groups. Alpha diversity was evaluated by three parameters: number of observed species (Observed), Shannon and Simpson indexes. These data were statistically analysed by Kruskal–Wallis' test followed by Dunn's post hoc test. Beta diversity was investigated by principal coordinate analysis (PCoA) with Bray‐Curtis dissimilarity and statistically analysed by PERMANOVA. To identify microbial biomarkers differentiating experimental groups, Linear Discriminant Analysis Effect Size (LEfSe) algorithm as implemented in the microeco R package (v1.15.0) was applied. LEfSe integrates non‐parametric statistics (Kruskal–Wallis and pairwise Wilcoxon tests) with Linear Discriminant Analysis to assess both statistical significance and biological relevance of taxa. The analysis was performed on normalized relative abundances, and taxa with an LDA score (log₁₀) greater than 2.0 and a *p*‐value below 0.05 were considered discriminant features. The resulting taxa were annotated according to the group in which they were most abundant, representing potential microbial biomarkers associated with specific host or conditions. Functional analysis was done using the FAPROTAX procaryotic database. These statistical analyses were firstly applied on the initial cohort (baseline) considering as discriminant factor “Colonization + Gram”, then they were applied on data recorded at three different times (T0, T1 and T2) considering “Colonization + Sepsis + Time” as factors.

### 2.4 Quantitative PCR Analysis and Data Processing

Quantitative PCR (qPCR) was performed to quantify *Escherichia/Shigella*, *Enterococcus faecium,* and *Peptoniphilus lacrimalis* in genomic DNA of randomly chosen 34 samples. Reactions were carried out using PowerUp SYBR Green Master Mix (Thermo Fisher Scientific, Milan, Italy) according to the manufacturer's instructions. Primers specific for each target species (*TEcol553/TEcol754*, *Ent1/Ent2*, *LACR/1392B*) and for the 16S rDNA gene (*fD1*/*rD1*) were used for amplification. Primer sequences and amplicon sizes were reported in Table 1.

**Table 1:** Primer used for qPCR validation of the significant species present in the signature.

Primer	Sequence (5′ → 3′)	Target/Organism	Amplicon size (bp)	Reference
LACR	GGCTTGACATATAAGAGACGAACT	*Pepetoniphilus lacrimalis*	420	Song et al. 2003
1392B	ACGGGCGGTGTGTAC	Eubacteria (16S rRNA)	—	Song et al. 2003
TEcol553 (tuf)	TGGGAAGCGAAAATCCTG	*Escherichia/Shigella*	258	Maheux et al. 2009
TEcol754 (tuf)	CAGTACAGGTAGACTTCTG	*Escherichia/Shigella*	258	Maheux et al. 2009
Ent1	TACTGACAAACCATTCATGATG	*Enterococcus faecium*	112	Ke et al. 1999
Ent2	AACTTCGTCACCAACGCGAAC	*Enterococcus faecium*	112	Ke et al. 1999

qPCR reactions were performed in a final volume of 10 µL containing 5 µL of master mix, primers at a final concentration of 10 µM each, and 4.5 µL of DNA template or standard. Amplification was carried out on a CFX96 Touch Real‐Time PCR Detection System (Bio‐Rad, Milan, Italy) under the following cycling conditions: initial denaturation at 94°C for 5 min, followed by 40 cycles of denaturation at 94°C for 1 min, annealing at 60°C for 1 min and extension at 72°C for 2 min and 30 s. A melt curve analysis was performed at the end of each run (60°C–95°C, 0.1°C/s increment) to confirm amplification specificity. Each sample was analysed in technical triplicate, and no‐template controls were included in every run.

The total bacterial 16S rDNA gene was used as the endogenous control for normalization and amplified using universal primers. Ct values were determined using the qPCR instrument software with automatic threshold settings. Relative quantification of bacterial species was calculated using the ΔCt method (ΔCt = Ct_species − Ct_16S). Results were expressed as 2^−ΔCt, representing the relative abundance of each bacterial species normalized to the total bacterial population (16S rDNA gene). Data were independently normalized using min–max scaling to a 0–1 range and subsequently visualized as a heatmap. Discrepancies between normalized qPCR measurements and normalized values obtained from the species abundance table were quantified and reported as percentage differences for each individual species.

## 3 Results

### 3.1 Cohort Description

A total of 132 patients were included in the study, of whom 94 were classified as Not Colonized and 38 as Colonized. There were 85 patients aged 65 or older and 49 patients younger than 65. Multidrug‐resistant (MDR) strains were identified in stool samples of septic patients at two timepoints (T0 and T1) and compared with the etiological agents isolated from blood cultures (Table 2). At T0, the most frequently detected MDR strain was *Escherichia coli* (57%), isolated predominantly in patients from the RIA (Anaesthesia and Intensive Care) and Internal Medicine departments. Other MDR strains included *Enterococcus faecium* (15%), *Pseudomonas aeruginosa* (14%), and co‐colonization with *P. aeruginosa* and *Klebsiella pneumoniae* KPC (14%). The most common bloodstream pathogens at this timepoint included *K. pneumoniae*, *Staphylococcus aureus*, *Staphylococcus epidermidis*, and *Enterococcus faecalis*.

**Table 2:** Colonization and etiological agents of sepsis.

MDR^1^ strain	Department	Etiological agent of sepsis
T0		
* Escherichia coli* (57%)	RIA^2^, IM^3^	*Klebsiella pneumoniae* or *Staphylococcus aureus* or *S. epidermidis*
* Enterococcus faecium* (15%)	RIA	*Enterococcus foecalis*
* Pseudomonas aeruginosa* (14%)	IM	*Streptococcus pyogenes*
* P. aeruginosa* + *K. pneumoniae* KPC (14%)	RIA	*K. pneumoniae* KPC
T1		
* E. coli* (18%)	CCHI^4^	*S. epidermidis*
* P. aeruginosa* (18%)	CCHI	*Klebsiella oxytoca* or *Proteus mirabilis* + *Streptococcus anginosus*
* P. aeruginosa* + *K. pneumoniae* (10%)	CCHI	*Serratia marcescens*
* K. pneumoniae* (27%)	RIA	*S. aureus* or *Klebsiella aerogenes* or *Staphylococcus hominis*
* K. pneumoniae* NDM (27%)	RIA	*E. coli* or *K. pneumoniae* NDM or *P. aeruginosa* + *Streptococcus gallolyticus*

*Note:* This table summarizes the MDR bacterial strains identified in stool samples from septic patients at baseline (T0) and follow‐up (T1), their hospital departments of origin (RIA: Reparto di Anestesia e Rianimazione; IM: Internal Medicine; CCHI: Chirurgia), and the etiological agents isolated from blood cultures during sepsis episodes. At T0, the most frequently identified MDR strain was *Escherichia coli* (57%), followed by *Enterococcus faecium* (15%) and *Pseudomonas aeruginosa* (14%). Mixed colonization with *P. aeruginosa* and *Klebsiella pneumoniae* KPC was also observed (14%). At T1, MDR isolates were more heterogeneous, with *K. pneumoniae* and *K. pneumoniae* NDM each accounting for 27% of the isolates, followed by *P. aeruginosa* (18%), *E. coli* (18%), and combinations thereof. Etiological agents of sepsis included Gram‐negative and Gram‐positive bacteria, with frequent co‐infections.

^1^ MD Departments: ^2^RIA: General Anaesthesia and Reanimation Unit; ^3^IM: Internal Medicine; ^4^CCHI: Cardio‐Thoracic‐Vascular Anaesthesia and Reanimation Unit of the Azienda Ospedaliera di Alessandria and Anaesthesia and Reanimation and Intensive Care Unit of the Ospedale Maggiore della Carità di Novara.

At T1, the distribution of MDR strains became more heterogeneous. *K. pneumoniae* and *K. pneumoniae* NDM were each identified in 27% of the patients, primarily in the RIA department. *P. aeruginosa* and *E. coli* were each detected in 18% of cases, while 10% showed co‐colonization with *P. aeruginosa* and *K. pneumoniae*. Blood cultures from these patients revealed a variety of etiological agents, including *S. epidermidis*, *K. oxytoca*, *Proteus mirabilis*, *Streptococcus anginosus*, *Serratia marcescens*, *Staphylococcus hominis*, and *Streptococcus gallolyticus*.

In the subgroup of patients with confirmed bloodstream infections, targeted antibiotic therapy was implemented in 38% of cases, while 57% received only empirical treatment. Infectious disease consultation was documented in 43% of these cases, although its timing and influence on therapy were variable. In contrast, among patients with negative blood cultures, none received targeted treatment, while empirical antibiotics were administered in 94%, often without infectious disease input. Among culture‐positive patients, the most administered antibiotics were piperacillin with β‐lactamase inhibitor (used in 76% of cases), daptomycin (29%), vancomycin (24%), meropenem (19%), amoxicillin with β‐lactamase inhibitor (19%), and linezolid (19%). Less frequently employed agents included ceftazidime (± inhibitor), cefotaxime, rifampicin, fosfomycin, oxacillin, and clindamycin.

### 3.2 Gut Microbiota Analysis

A total of 6,537,595 reads (with a mean value of 49,905 reads per sample), after the demultiplexing step, were used for biodiversity analysis. The genomic sequences were included in the BioProject PRJNA1125274 available in NCBI database and contained 289 BioSamples.

#### 3.2.1 Baseline Characterization of Gut Microbiota

**Figure 1:**
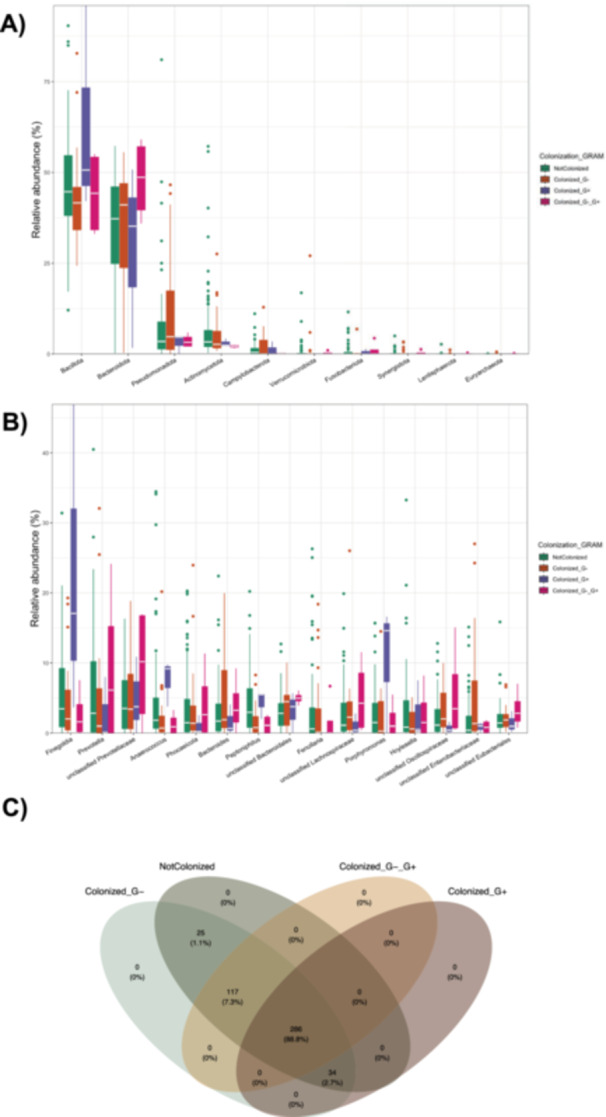
Distribution of taxa according to bacterial colonization status. The figure illustrates the relative frequencies of four colonization categories identified in the study population: Not Colonized, Colonized with Gram‐negative bacteria (Colonized_G−), Colonized with both Gram‐negative and Gram‐positive bacteria (Colonized_G−_G+), and Colonized with Gram‐positive bacteria only (Colonized_G+). A total of 132 patients were included in the study, of whom 94 were classified as Not Colonized and 38 as Colonized. There were 85 patients aged 65 or older and 49 patients younger than 65. A total of 289 sequences were used for statistically comparison. (A) Relative abundance at phylum level; (B) relative abundance at genus level; (C) Venn diagram showing overlap of OTUs among categories.

Bacterial colonization patterns at the basal level were assessed across all samples and categorized into four groups based on the presence of Gram‐negative and/or Gram‐positive bacteria colonization (Figure 1). The assessment of the relative abundance of both phyla and genera associated with different colonisation of patients at T0 (baseline at admission) showed substantial homogeneity in the composition of the microbiota in the different categories of patients. In fact, no significant differences were evident. More in detail, Figure 1A illustrates the relative abundances of bacterial phyla across the four study categories (Not colonized, Gram‐negative, Gram‐positive, and both). Across all groups, *Bacillota* and *Bacteroidota* dominate the microbial profiles, accounting for approximately 80%–95% of the community composition. At the genus level (Figure 1B), a predominance of strictly anaerobic genera, including *Finegoldia*, *Prevotella*, *Anaerococcus*, *Peptoniphilus*, *Phocaeicola*, *Fenollaria* (the only one significant among the different groups, *p* value 0.01641), *Hoylesella*. These taxa were detected across all groups, with a wide variability in abundance. Although slight enrichment of *Finegoldia*, *Anaerococcus, Peptoniphylus,* and *Porphyromonas* was observed in the G+ group, no statistically significant differences in genus‐level abundance were detected among colonization categories. On the contrary, the genus *Fenollaria* was absent in colonized G+.

The Venn diagram (Figure 1C) illustrates the overlap of detected genera among the four groups. A total of 286 (88.8%) OTU were shared across all categories, forming the common core microbiota.

34 OTUs were common to the NotColonized, Colonized_G− and Colonized_G+ groups. 117 OTUs were present in the NotColonized, Colonized_G− and Colonized_G−_G+ groups. Finally, 25 OTUs were present in both NotColonized and Colonized_G−.

**Figure 2:**
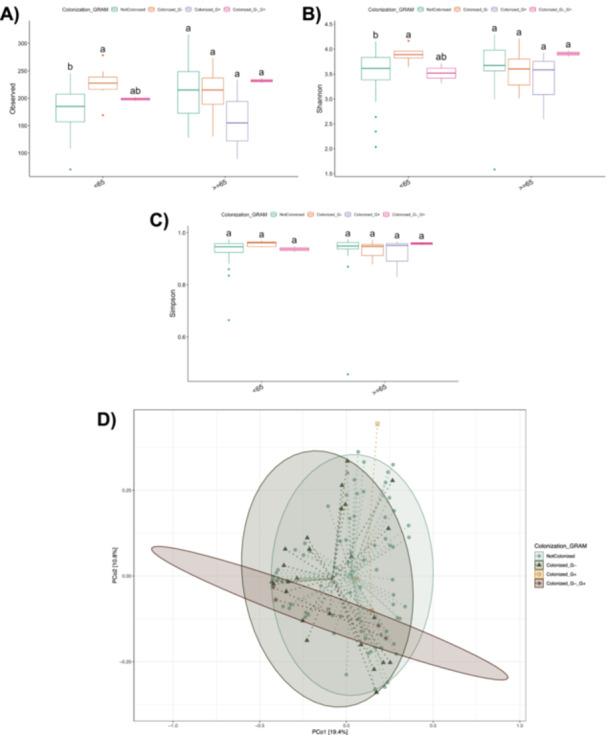
Alpha and beta‐ diversity according to colonization status. (A–C) Alpha diversity analysis considering Colonization_gram category. Bars topped by the same letter within each parameter do not differ significantly at *p* < 0.05. (A) Observed: number of observed species in each considered category. (B and C) Shannon and Simpson index respectively in each considered category. (D) Beta diversity‐ Principal Coordinates Analysis (PCoA) of microbial community composition according to colonization status. Ordination plots illustrate the distribution of samples along the first two principal coordinates (PCo1 and PCo2). Samples are grouped by colonization category: Not Colonized, Colonized with Gram‐negative bacteria (Colonized_G−), Colonized with Gram‐positive bacteria (Colonized_G+), and Colonized with both Gram‐negative and Gram‐positive bacteria (Colonized_G−_G+). Each point represents an individual sample, labeled according to colonization status. A total of 132 patients were included in the study, of whom 94 were classified as Not Colonized and 38 as Colonized. There were 85 patients aged 65 or older and 49 patients younger than 65. A total of 289 sequences were used for statistically comparison.

Alpha diversity indices were compared across colonization categories and are presented as box plots in Figures 2A–C. Notably, alpha diversity was stratified by age group to explore potential age‐related effects. Samples collected from younger individuals demonstrated lower alpha diversity compared to older subjects, regardless of colonization status. This difference was statistically significant indicating that age may also influence the diversity of the gut microbiota.

Principal Coordinates Analysis (PCoA) was performed to assess differences in microbial community composition among the four colonization groups (beta‐diversity). The ordination plot (Figure 2D) shows clear clustering of samples along the first two principal coordinates, which together accounted for 30.2% of the total variance in microbial profiles (PCo1: 19.4%, PCo2: 10.8%).

Samples classified as Colonized with Gram‐positive bacteria (Colonized_G+) were distributed separately from those colonized with Gram‐negative bacteria (Colonized_G−) and from the Not Colonized group, indicating distinct microbial community structures associated with Gram‐positive colonization. In contrast, samples co‐colonized by both Gram‐negative and Gram‐positive bacteria (Colonized_G−_G+) appeared more dispersed across the ordination space, partially overlapping with the other groups. To statistically validate these differences, a PERMANOVA test was performed, but no statistical significance among colonization groups (*p*‐value = 0.156) was detected.

**Figure 3:**
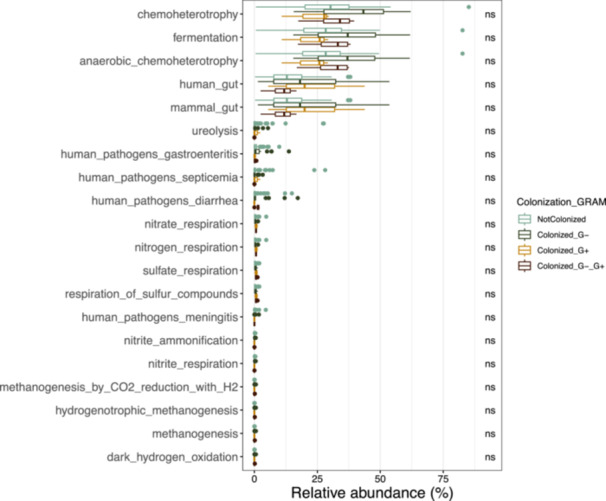
Functional potential of microbial communities under different colonization states. Relative abundance (%) of predicted metabolic pathways and functional traits in samples stratified by colonization pattern: non‐colonized (NotColonized), colonized by Gram‐negative bacteria (Colonized_G−), Gram‐positive bacteria (Colonized_G+), or both (Colonized_G−_G+). Predicted functions include core anaerobic and respiratory pathways (e.g., methanogenesis, sulfate respiration, nitrate respiration), nitrogen and sulfur cycling processes, and traits linked to host adaptation such as mammalian gut or human gut signatures. Categories related to pathogenicity (e.g., human pathogens associated with meningitis, diarrhea, septicemia) are also displayed. Bars represent mean relative abundance of each functional category within groups. “ns” indicates non‐significant differences between groups.

To explore the functional potential of microbial communities associated with different colonization states, we compared the relative abundance of predicted metabolic pathways across five groups: non‐colonized (NotColonized), colonized by Gram‐negative bacteria (Colonized_G−), colonized by Gram‐positive bacteria (Colonized_G+), colonized by both Gram‐negative and Gram‐positive bacteria (Colonized_G −_G+), and an aggregated colonization category (Colonization_GRAM). FAPROTAX‐derived functional predictions indicated statistically significant differences in core anaerobic processes and host‐associated traits between colonization states (Figure 3). Methanogenesis‐related pathways, including hydrogenotrophic methanogenesis and methanogenesis by CO₂ reduction with H₂, were consistently more abundant in colonized groups compared to the non‐colonized state, although differences did not reach statistical significance (ns). Similarly, pathways involved in anaerobic chemoheterotrophy and fermentation dominated across all groups, reflecting the adaptation of these communities to low‐oxygen environments. Nitrogen and sulfur cycling functions showed variable enrichment: nitrate and nitrite respiration were more frequently associated with Gram‐negative colonization, whereas sulfate respiration and sulfur compound utilization were detected at higher levels in samples colonized by both Gram‐positive and Gram‐negative bacteria. Signatures related to mammalian and human gut adaptation were more pronounced in colonized states, particularly in the combined Gram‐positive and Gram‐negative group, supporting a functional shift toward host‐associated traits. Interestingly, predicted pathogenic potential (e.g., functions linked to meningitis, septicaemia, and gastroenteritis) was present at low relative abundance and showed no significant differences between groups.

#### 3.2.2 Temporal Dynamics in Microbial Composition in Gut Microbiota

**Figure 4:**
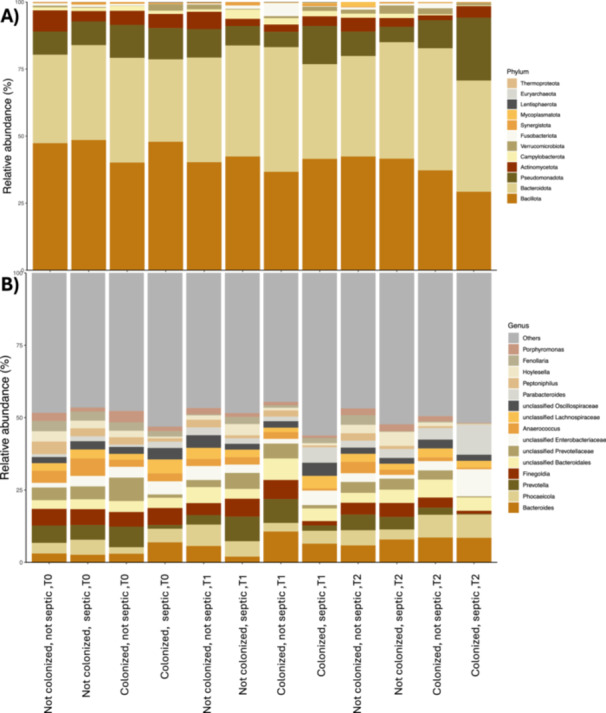
Temporal changes in microbial community composition by colonization and septic status. (A) Relative abundance of major bacterial phyla across samples stratified by colonization status (colonized vs. Not Colonized) and presence or absence of sepsis (septic vs. not septic) at three time points (T0, T1, T2). Bars topped by the same letter within each parameter do not differ significantly at *p* < 0.05. (B) Relative abundance of the main bacterial genera detected under the same conditions. Each bar represents the median relative abundance per group. A total of 132 patients were included in the study, of whom 94 were classified as Not Colonized and 38 as Colonized. There were 85 patients aged 65 or older and 49 patients younger than 65. A total of 289 sequences were used for statistically comparison.

To assess temporal dynamics in microbial composition according to colonization status and septic condition, relative abundance profiles at the phylum (Figure 4A) and genus level (Figure 4B) across three time points (T0, T1, T2) were analyzed. At phylum level, *Bacillota* and *Bacteroidota* dominated across all groups, accounting together for most detected taxa. *Pseudomonadota* and *Actinomycetota* were consistently present at lower relative abundances, while minor phyla such as *Fusobacteriota*, *Campylobacterota*, and *Verrucomicrobiota* were sporadically detected. Archaeal signatures (*Thermoproteota*, *Euryarchaeota*) appeared at very low abundance (< 1%). The overall phylum‐level structure remained relatively stable over time, although samples from colonized septic patients exhibited a modest increase in *Bacillota* (*p*‐value 0.003715) compared to non‐septic individuals.

At the genus level, compositional heterogeneity was more evident. Core genera included *Bacteroides*, *Phocaeicola*, and *Prevotella*, which were more abundant in non‐septic samples. Moreover, genera associated with opportunistic or pathogenic traits—such as *Finegoldia*, *Anaerococcus*, and *Peptoniphilu*s—also showed an enrichment in colonized, not septic patients, particularly at T1 and T2. Members of unclassified *Lachnospiraceae* and *Oscillospiraceae* were consistently present but did not display marked temporal shifts.

**Figure 5:**
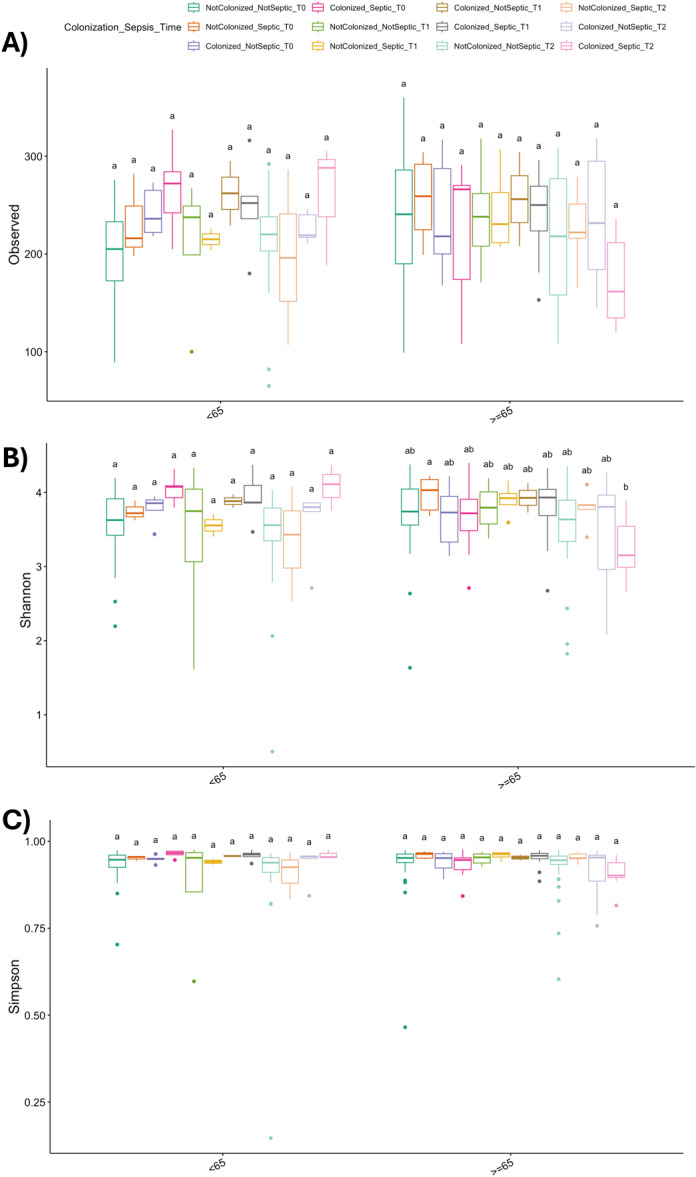
Gut microbiota alpha diversity in septic patients enrolled in the SURVEIL project. Alpha diversity metrics were compared between patient subgroups to evaluate the impact of sepsis on gut microbial diversity. (A) Observed species richness, (B) Shannon diversity index, and (C) Simpson diversity index. Statistical differences were assessed using the Wilcoxon rank‐sum test; boxes represent interquartile ranges, and horizontal lines denote medians. Asterisks indicate statistical significance (*p* < 0.05). A total of 132 patients were included in the study, of whom 94 were classified as Not Colonized and 38 as Colonized. There were 85 patients aged 65 or older and 49 patients younger than 65. A total of 289 sequences were used for statistical comparison.

Analysis of gut microbiota alpha diversity revealed a reduction in microbial diversity among septic patients (Figure 5A). As shown in Figure 5A, the Observed species richness was lower in the sepsis group compared to controls in the older subjects, indicating a loss of microbial complexity, even if not statistically significant. Similarly, the Shannon index (Figure 5B) and the Simpson index (Figure 5C) were both significantly reduced in older septic patients, reflecting not only diminished richness but also altered evenness of the microbial community. These findings suggest a profound dysbiosis associated with sepsis in older patients. The differences were statistically significant.

**Figure 6:**
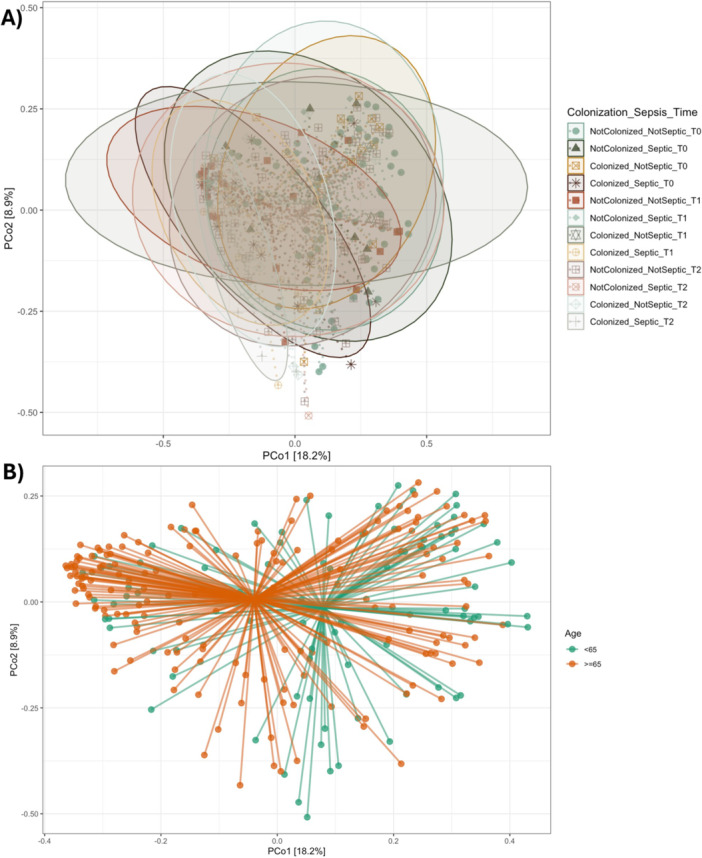
Principal Coordinates Analysis (PCoA) of gut microbiota composition across colonization and sepsis status (A) and age‐related differences in gut microbiota composition visualized by PCoA (B). PCoA plot based on Bray–Curtis dissimilarity illustrating beta diversity differences in gut microbial communities among groups defined by colonization status (colonized vs non‐colonized) and sepsis condition (septic vs. non‐septic) at timepoint T1. Each point represents a stool sample, and groupings are visualized using distinct symbols and 95% confidence ellipses. A permutational multivariate analysis of variance (PERMANOVA) revealed a statistically significant difference in community structure among the groups (*F* = 1.70, *p* = 0.001, *R*² = 0.072), indicating that both sepsis and colonization status significantly influence gut microbiota composition. Principal Coordinates Analysis (PCoA) based on Bray–Curtis dissimilarity illustrates the beta diversity of gut microbial communities stratified by age (B): participants younger than 65 years (green) and those aged 65 years or older (orange). Each line connects an individual sample to the group centroid, representing dispersion within each age group. The first two principal coordinates explain 18.2% and 8.9% of the variance, respectively. The plot highlights differences in microbial community structure associated with aging.

To investigate the impact of colonization and sepsis on gut microbiota composition over time, beta diversity analyses were performed at timepoints T0, T1, and T2 (Figure 6A). Principal Coordinates Analysis (PCoA) revealed temporal changes in microbial community structure associated with colonization status (colonized vs. non‐colonized) and clinical condition (septic vs. non‐septic).

Microbial communities showed partial but clear clustering according to the combined variable Colonization_Sepsis_Time (Figure 6A). PERMANOVA analysis confirmed a statistically significant difference in beta diversity among the 12 groups (*F* = 1.70, *p*‐value = 0.001, *R*² = 0.072), indicating that colonization and sepsis status explained 27.1% of the variance in microbial composition at these timepoints. To assess the impact of age on gut microbiota composition, a beta diversity analysis was performed using Bray–Curtis dissimilarity, and the results were visualized through Principal Coordinates Analysis (PCoA). As shown in Figure 6B, samples from participants aged ≥ 65 years exhibited a distinct clustering pattern compared to those < 65 years. Each sample is represented by a vector originating from the group centroid, reflecting within‐group dispersion.

The first two principal coordinates explained 18.2% and 8.9% of the total variance, respectively. Overall, older individuals (≥ 65 years) showed increased intra‐group variability and a shift in microbial community structure compared to younger individuals (*p*‐value 0.002). These findings suggest that aging is associated with compositional alterations in the gut microbiota.

Similar trends were observed at T0 and T2, with PCoA plots showing group‐dependent dispersion patterns and divergence in community profiles. Although inter‐individual variability remained high, the results consistently support the hypothesis that gut microbiota composition is influenced by both colonization dynamics and septic status across the clinical timeline.

To identify bacterial taxa differentially enriched across clinical groups, we performed a LEfSe analysis based on colonization status, sepsis condition, and timepoint (Table 3). Several taxa showed significant differential abundance (adjusted *p* < 0.01, LDA > 2) across conditions.

**Table 3:** Discriminant bacterial taxa across colonization and sepsis status at different timepoints identified by LEfSe analysis.

Phylum	Species	Group	LDA score	Adjusted *p* value	Significance
Bacillota	*Ezakiella coagulans*	NotColonized_NotSeptic_T0	3.720206765	0.001145644	**
Bacillota	*Peptoniphilus lacrimalis*	NotColonized_NotSeptic_T0	3.64244687	8.50E‐05	***
Bacillota	*Anaerococcus tetradius*	NotColonized_NotSeptic_T0	3.58214301	0.004410526	**
Bacillota	*Ezakiella sp*.	NotColonized_NotSeptic_T0	3.136896593	0.00382704	**
Bacillota	*Peptoniphilus nemausensis*	NotColonized_NotSeptic_T0	3.081120647	0.000723553	***
Bacillota	*Peptoniphilus sp*.	NotColonized_NotSeptic_T0	2.96379065	5.90E‐05	***
Bacillota	*Peptoniphilus timonensis*	NotColonized_NotSeptic_T0	2.485996951	0.003692332	**
Bacillota	*Dialister sp*.	Colonized_NotSeptic_T0	3.124149335	0.001909684	**
Bacillota	*Peptoniphilus faecalis*	Colonized_NotSeptic_T0	2.474972455	0.006199027	**
Bacillota	*Peptoniphilus porci*	NotColonized_Septic_T0	3.442435212	0.004066425	**
Pseudomonadota	*unclassified Escherichia/Shigella*	Colonized_Septic_T0	2.925773299	0.003142962	**
Pseudomonadota	*Atlantibacter hermannii*	Colonized_Septic_T0	2.774951725	0.003733211	**
Bacillota	*unclassified Luoshenia*	Colonized_Septic_T0	2.21992878	0.003142962	**
Bacillota	*Finegoldia sp*.	Colonized_NotSeptic_T1	4.018396455	0.003733211	**
Bacillota	*Anaerococcus sp*.	Colonized_NotSeptic_T1	3.902895432	0.006048656	**
Bacillota	*Peptoniphilus harei*	Colonized_NotSeptic_T1	3.691195397	0.000305503	***
Bacillota	*Peptoniphilus gorbachii*	Colonized_NotSeptic_T1	3.420602773	4.82E‐06	***
Bacillota	*Peptoniphilus grossensis*	Colonized_NotSeptic_T1	3.263478586	0.000305503	***
Pseudomonadota	*Desulfovibrio piger*	Colonized_NotSeptic_T1	2.861832643	1.52E‐05	***
Actinomycetota	*Atopobium minutum*	Colonized_NotSeptic_T1	2.412539336	0.003142962	**
Bacteroidota	*Parabacteroides sp*.	Colonized_Septic_T1	3.532834812	0.000388259	***
Pseudomonadota	*Atlantibacter sp*.	Colonized_Septic_T1	2.917836144	0.000305503	***
Euryarchaeota	*Methanobrevibacter smithii*	Colonized_Septic_T1	2.272382804	0.006199027	**
Bacteroidota	*Hoylesella timonensis*	NotColonized_Septic_T1	4.23419728	0.00195225	**
Bacillota	*Dialister propionicifaciens*	NotColonized_Septic_T1	3.999660185	0.003142962	**
Bacteroidota	*Sodaliphilus sp*.	NotColonized_Septic_T1	3.051848682	0.003142962	**
Bacillota	*Enterococcus hirae*	Colonized_Septic_T2	2.818996153	0.002273404	**
Bacillota	*unclassified Phascolarctobacterium*	Colonized_Septic_T2	2.742543855	0.00776139	**
Bacillota	*Enterococcus faecium*	Colonized_Septic_T2	2.628919791	0.006048656	**

*Note:* Linear discriminant analysis effect size (LEfSe) was used to identify bacterial taxa differentially enriched across clinical groups defined by colonization status (colonized vs not colonized), sepsis condition (septic vs. non‐septic), and timepoints (T0, T1, T2). The analysis revealed several taxa significantly associated with specific group‐time combinations (adjusted *p* < 0.01, LDA score > 2).

Differentially abundant taxa identified by the LEfSe (Linear Discriminant Analysis Effect Size) method implemented in the microeco R package. The analysis was based on non‐parametric tests (Kruskal–Wallis and pairwise Wilcoxon) followed by LDA to estimate the effect size of each taxon across experimental groups.

Column definitions:

*Taxon* (*Phylum and Species*)—bacterial taxonomic unit showing differential relative abundance among groups.

*LDA_score* (log₁₀)—Linear Discriminant Analysis score quantifying the magnitude of difference in relative abundance between groups; higher absolute values indicate stronger discriminative power (typically threshold > 2.0 considered biologically relevant).

*p_value*—significance level from the Kruskal–Wallis or Wilcoxon test indicating whether abundance differences among groups are statistically significant (*p* < 0.05 considered significant).

*Group*—the category (experimental condition or treatment level) in which the corresponding taxon is most abundant and statistically enriched, representing the group‐specific biomarker.

*Adjusted_p_value*—*p*‐value corrected for multiple testing (e.g., using Benjamini–Hochberg FDR adjustment).

At T0, the NotColonized_NotSeptic group was enriched in several *Bacillota* species, including *Ezakiella coagulans*, *Peptoniphilus lacrimalis*, and *Anaerococcus tetradius*. In contrast, the Colonized_NotSeptic group showed an over representation of unclassified *Prevotellaceae*, *Dialister* sp., and *Peptoniphilus faecalis*. Within the Colonized_Septic group, key discriminant taxa included unclassified *Escherichia/Shigella* and *Atlantibacter hermannii* suggesting a shift toward potentially opportunistic or pathobiont‐enriched communities.

At T1, the Colonized_NotSeptic group showed strong enrichment in multiple *Peptoniphilus* species (*P. harei*, *P. gorbachii*, *P. grossensis*), as well as *Anaerococcus* sp., *Finegoldia* sp., and *Desulfovibrio piger*. Conversely, the Colonized_Septic group exhibited higher levels of *Parabacteroides* sp., *Atlantibacter* sp., and the archaeon *Methanobrevibacter smithii*. In the NotColonized_Septic group, differentially abundant taxa included *Hoylesella timonensis*, *Dialister propionicifaciens*, and *Sodaliphilus* sp.

By T2, discriminatory taxa remained detectable in the Colonized_Septic group, which was enriched in *Enterococcus hirae*, *Enterococcus faecium*, and unclassified *Phascolarctobacterium*, suggesting persistence of dysbiotic signatures over time.

#### 3.2.3 qPCR‐Based Validation of Microbial Targets

**Figure 7:**
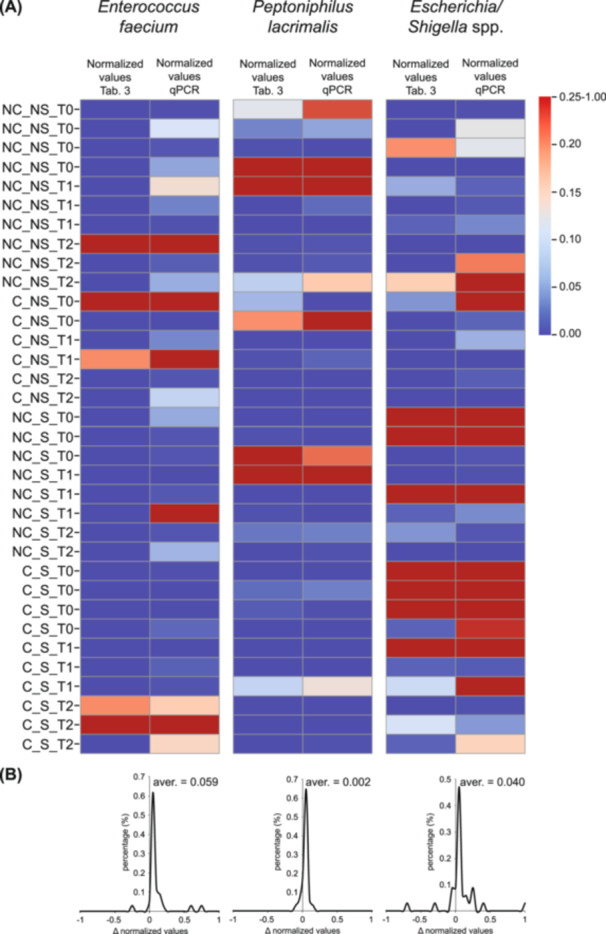
qPCR validation of species abundance (Table 3). (A) Data from 34 samples of *Enterococcus faecium*, *Peptoniphilus lacrimalis*, and *Escherichia/Shigella* spp., derived from the species abundance table (Table 3) and qPCR analysis, were independently normalized on a scale from 0 to 1 and visualized using a heatmap. (B) The differences between normalized qPCR values and normalized values from the species abundance table show a distribution with a mean close to zero, indicating strong concordance and significant validation. C, colonized; NC, not colonized; NS, not septic; S, septic; T0, time of enrollment; T1, time of diagnosis; T2, time of discharge or death.

qPCR validation was performed on 34 longitudinal samples to independently assess the abundance of *Enterococcus faecium*, *Peptoniphilus lacrimalis*, and *Escherichia/Shigella* spp., previously identified in the species abundance table (Table 3). For each taxon, values derived from amplicon sequencing (Table 3) and qPCR were independently normalized on a 0–1 scale to allow direct comparison across samples and time points (T0, T1, T2), stratified by colonization (C vs. NC) and sepsis status (S vs. NS). As shown in Figure 7A, the heatmap comparison revealed a high degree of concordance between normalized sequencing‐based abundances and qPCR‐derived values across all three taxa. For *E. faecium*, samples exhibiting high relative abundance in Table 3 consistently showed elevated normalized qPCR values, particularly in colonized septic (C_S) individuals at later time points. Similarly, *P. lacrimalis* displayed overlapping intensity patterns between the two datasets, with increased normalized values in selected NotColonized_Septic (NC_S) and Colonized_NotSeptic (C_NS) samples, reflecting a coherent detection trend. For *Escherichia/Shigella* spp., concordant high‐abundance signals were observed predominantly in septic groups, with both methods identifying comparable peaks across matched samples.

To quantitatively evaluate agreement between methods, the difference (Δ) between normalized qPCR values and normalized sequencing‐derived values for each sample was calculated (Figure 7B). The distribution of Δ values was centered close to zero for all taxa, with mean differences of 0.059 for *E. faecium*, 0.002 for *P. lacrimalis*, and 0.040 for *Escherichia/Shigella* spp. These near‐zero averages indicate minimal systematic bias between methodologies. The majority of Δ values clustered tightly around zero, with only limited dispersion toward positive or negative extremes, supporting strong methodological concordance.

## 4 Discussion

This study provides an integrated view of gut microbiota dynamics, bacterial colonization, and multidrug‐resistant (MDR) organism carriage in a hospitalized cohort, with particular emphasis on the interplay between age, sepsis, and microbial ecology. At baseline, most patients were not colonized, whereas a minority harbored Gram‐negative, Gram‐positive, or mixed bacterial populations. The predominance of *Escherichia coli*, *E. faecium*, and *Pseudomonas aeruginosa* among MDR strains, particularly in patients admitted to intensive care and internal medicine units, reflects the well‐documented nosocomial reservoir of these pathogens (Peleg and Hooper 2010; Magill et al. 2014). The emergence of *Klebsiella pneumoniae* and carbapenemase‐producing variants (KPC, NDM) at subsequent timepoints highlights the adaptive capacity of opportunistic Enterobacterales to persist and disseminate under antibiotic pressure (Nordmann et al. 2011). The heterogeneity of bloodstream isolates observed in this study further emphasizes the multifactorial nature of infection onset, where colonization may represent a predisposing but not the exclusive factor (Donskey 2004; Taur et al. 2012).

Despite the presence of MDR strains, the baseline microbiota composition remained relatively homogeneous across colonization groups. *Bacillota* and *Bacteroidota* dominated all profiles, consistent with the established configuration of the healthy intestinal microbiota (Qin et al. 2010). The detection of *Finegoldia* sp., *Prevotella* sp., *Anaerococcus* sp., and *Peptoniphilus* sp. as core genera across all groups indicates the persistence of obligate anaerobes typical of low‐oxygen environments, even in clinically fragile patients. These taxa are known to contribute to short‐chain fatty acid (SCFA) production and mucosal integrity (Louis and Flint 2017). Although *Fenollaria* sp. showed group‐specific differences—being absent in Gram‐positive colonized samples—overall genus‐level variability was limited, supporting the notion of a resilient microbial core despite clinical heterogeneity (Lozupone et al. 2012).

The Venn diagram analysis corroborated this stability, revealing that over 88% of operational taxonomic units (OTUs) were shared across all colonization categories, suggesting that colonization involves modulation of existing taxa rather than the introduction of entirely novel microorganisms. This finding aligns with prior studies reporting that hospital‐associated dysbiosis frequently manifests as altered abundance patterns within a pre‐existing microbial framework (Zaborin et al. 2014; Taur et al. 2012).

Microbial alpha diversity further reflected this ecological resilience. No significant differences were detected between colonization states, suggesting that, at the time of admission, MDR colonization *per se* did not translate into measurable alterations in global richness or evenness. In contrast, age emerged as a major determinant of community structure. Older patients displayed significantly higher alpha diversity compared with younger individuals, supporting the concept of age‐associated diversification and ecological stabilization of the gut microbiota described in elderly cohorts (Claesson et al. 2012; O'Toole and Jeffery 2015). Longitudinal and cross‐sectional studies have consistently shown that, after adulthood, the gut microbiome may reach a relatively stable and individualized configuration, often characterized by expanded phylogenetic breadth and functional redundancy (Falony et al. 2016; Jackson et al. 2018). This increased complexity has been interpreted as a marker of ecological maturity and resilience, although it may coexist with inflammaging‐related functional shifts (Franceschi et al. 2018; Ghosh et al. 2020).

In contrast, younger subjects in our cohort exhibited reduced alpha diversity, potentially reflecting less consolidated microbial networks or a greater susceptibility to perturbations associated with hospitalization, antimicrobial exposure, or acute illness. Reduced richness and evenness have been repeatedly associated with ecological fragility and increased vulnerability to dysbiosis in both pediatric and young adult settings (Yatsunenko et al. 2012). Moreover, hospital‐related stressors—including antibiotic administration, dietary modifications, and environmental microbial exposure—can disproportionately affect communities with lower baseline redundancy, leading to more pronounced compositional instability (Dethlefsen and Relman 2011; Zaborin et al. 2014).

Importantly, the absence of alpha diversity differences between colonized and non‐colonized patients aligns with previous observations indicating that MDR colonization may occur within microbiota configurations that remain globally diverse but are ecologically permissive to specific taxa (Buffie and Pamer 2013; Taur and Pamer 2014). In this context, colonization resistance is not solely dependent on overall diversity but rather on specific compositional and functional attributes, including niche occupancy, metabolite production, and competitive exclusion dynamics (Kamada et al. 2013; Pickard et al. 2017). Therefore, preserved alpha diversity does not necessarily equate to preserved colonization resistance, particularly in the setting of hospitalization and systemic inflammation.

Collectively, these findings emphasize that host‐related variables—most notably age—may outweigh colonization status in shaping the baseline ecological structure of the gut microbiome at admission. Age‐related microbial architecture likely reflects long‐term host–microbe coadaptation and cumulative environmental exposures, thereby exerting a stronger influence on richness and evenness metrics than the presence of MDR colonizers alone. This reinforces the need to consider demographic and host intrinsic factors as primary ecological drivers when interpreting microbiome data in clinical cohorts, particularly in studies investigating colonization and sepsis risk.

Beta diversity analyses revealed subtle but biologically meaningful differences. Samples colonized by Gram‐positive bacteria clustered apart from those colonized by Gram‐negative organisms and from non‐colonized patients, suggesting compositional divergence related to microbial physiology or ecological niche preference (Lozupone et al. 2012). Nevertheless, PERMANOVA testing indicated that these differences were not statistically significant, consistent with prior findings that early colonization produces limited shifts in overall microbial community architecture (Taur et al. 2012). The broader dispersion observed among co‐colonized samples likely reflects increased ecological heterogeneity resulting from interactions between distinct bacterial guilds and antibiotic selection pressure.

From a functional perspective, predicted metagenomic profiles obtained using FAPROTAX, suggested that metabolic potential remained largely consistent across colonization categories. Core anaerobic functions such as fermentation and chemoheterotrophy were conserved, in agreement with the physiological adaptation of gut microbiota to anoxic conditions (Louis and Flint 2017). Methanogenesis‐related pathways were more pronounced in colonized patients, particularly those harbouring both Gram‐positive and Gram‐negative taxa, possibly indicating enhanced reductive metabolism under dysbiosis conditions. Likewise, modest enrichment of nitrate and nitrite respiration in Gram‐negative colonization and of sulfate reduction in mixed colonization states suggests redox imbalances that may favour opportunistic taxa (Zaborin et al. 2014). Importantly, the predicted abundance of pathogenicity‐associated functions (e.g., genes linked to septicemia or meningitis) remained low and did not differ between groups, implying that colonization does not necessarily equate to functional virulence activation. Moreover, the associations observed between microbiota composition, colonization status, and sepsis should be interpreted cautiously. The temporal analysis revealed that, over time, the overall phylum‐level structure remained stable, dominated by *Bacillota* and *Bacteroidota*, while *Pseudomonadota* and *Actinomycetota* persisted at lower relative abundances. Nevertheless, older septic patients displayed a significant reduction in microbial diversity, particularly in Shannon and Simpson indices, consistent with loss of evenness and resilience (Taur et al. 2012; Zaborin et al. 2014). The enrichment of opportunistic taxa such as *Finegoldia* sp., *Anaerococcus* sp., and *Peptoniphilus* sp. in colonized non‐septic patients suggests that colonization may precede systemic infection by establishing a permissive microbial niche, as also proposed in previous models of pre‐sepsis dysbiosis (Alverdy and Krezalek 2017). Although partial overlap was observed between MDR colonizers and bloodstream isolates, this finding should not be interpreted as direct evidence of intestinal translocation. The relationship between colonization and infection is complex and may reflect ecological dominance, antibiotic‐driven selection, or exposure to shared hospital reservoirs rather than a direct causal pathway. Colonization with MDR organisms is widely recognized as a risk factor for infection, but it does not necessarily predict the infecting strain or its anatomical source. Alternative explanations—including antibiotic selective pressure, ICU environmental ecology, and host susceptibility—likely contribute to the observed concordance. These interpretations are consistent with previous work showing that intestinal domination increases infection risk without invariably determining pathogen identity (Taur et al. 2012; Donskey 2004).

Beta diversity comparisons across colonization and sepsis categories confirmed that microbial composition was jointly influenced by these clinical variables, explaining a measurable proportion of variance (PERMANOVA *R*² = 0.072, *p* = 0.001). Such patterns support the concept of interactive ecological drivers, where colonization and sepsis act synergistically to modulate microbial community organization (Belkaid and Hand 2014). Age also contributed to these compositional shifts, with older individuals exhibiting distinct clustering patterns and greater within‐group variability—hallmarks of reduced microbiome stability (O'Toole and Jeffery 2015).

Finally, LEfSe analysis identified specific bacterial biomarkers discriminating between colonization and sepsis states across timepoints. Enrichment of *Escherichia/Shigella* and *Atlantibacter hermannii* in colonized septic patients at T0–T1, and persistence of *Enterococcus faecium* and *E. hirae* at T2, reflect sustained selection for facultative anaerobes with pathogenic potential. Conversely, non‐septic colonized patients were enriched in *Peptoniphilus* sp., *Finegoldia* sp., and *Anaerococcus* sp., suggesting compensatory proliferation of obligate anaerobes that may maintain partial ecological balance (Louis and Flint 2017). These findings support a two‐stage model of dysbiosis, wherein colonization represents an early adaptive shift that precedes deeper microbial disruption during sepsis progression (Alverdy and Krezalek 2017; Shimizu et al. 2020b).

Collectively, this work demonstrates that colonization, age, and sepsis interact to shape the gut microbiota's ecological and functional landscape. The persistence of a shared microbial core underscores the microbiota's resilience, but age‐associated loss of diversity and selective enrichment of opportunistic taxa signal diminished stability and greater vulnerability to infection. Overall, the consistency between sequencing‐based relative abundance estimates and qPCR quantification confirms the robustness of the taxonomic signals reported and supports the validity of the identified species‐level trends across colonization status, sepsis condition, and longitudinal sampling points. Future studies integrating shotgun metagenomics and metabolomics will be instrumental to clarify how these microbial transitions influence host physiology, antibiotic response, and sepsis outcomes. Ultimately, the identification of microbial biomarkers such as *Escherichia/Shigella* and *Enterococcus faecium*, also validated by qPCR results, may guide the development of predictive and preventive microbiome‐based strategies in high‐risk hospitalized populations (Taur et al. 2012; Zaborin et al. 2014; Claesson et al. 2012).

### 4.1 Study Limitations

This study has several limitations that should be acknowledged. First, the use of 16S rDNA sequencing limits taxonomic resolution to the genus or, in some cases, species level and does not provide direct insight into microbial gene content or activity. Although, predictive functional profiling was performed, the obtained findings do not establish causality but rather identify ecological patterns that may generate mechanistic hypotheses. The detected shifts may reflect consequences of systemic inflammation, antibiotic exposure, or critical illness–related physiological changes rather than primary drivers of sepsis onset. Future studies integrating shotgun metagenomics, metabolomics, and host immunological profiling will be required to determine whether the microbial signatures, identified here, play an active role in disease pathogenesis or represent secondary ecological responses. Second, while the longitudinal design allowed us to assess temporal microbiota changes, sampling was limited to three discrete timepoints (admission, sepsis onset, and discharge), potentially missing dynamic microbial fluctuations in response to clinical interventions such as antibiotic therapy or changes in nutritional support. Third, although the studied cohort was prospectively enrolled and clinically well characterized, sample size—especially within certain subgroups—may have limited statistical power to detect more subtle associations or interaction effects (e.g., between specific MDR strains and microbial shifts). Additionally, the study was conducted in two hospitals within a specific regional healthcare context, which may limit generalizability to other settings or populations. Lastly, the observational nature of the study precludes causal inference; while the authors observed significant associations between colonization, microbiota changes, and sepsis, further mechanistic studies are needed to elucidate causal pathways and test microbiota‐targeted interventions.

## Author Contributions

**Chiara Bazzano:** investigation, data curation, formal analysis, writing – original draft, writing – review and editing. **Alice Caramaschi:** data curation, investigation, formal analysis, writing – original draft, writing – review and editing. **Nadia Massa:** software, data curation, writing – original draft, writing – review and editing. **Silvio Collani:** formal analysis, validation, writing – review and editing. **Marta Mellai:** formal analysis, writing – original draft, writing – review and editing. **Nadia Barizzone:** formal analysis, methodology, writing – review and editing. **Andrea Rocchetti:** conceptualization, investigation, supervision, writing – review and editing. **Paolo Bottino:** formal analysis, data curation. **Maria Simona Caroppo:** formal analysis and data curation. **Valeria Bonato:** formal analysis and data curation. **Rosanna Vaschetto:** formal analysis and data curation. **Sara Scaglione:** data curation, formal analysis, and investigation. **Alessia Francese:** conceptualization and investigation. **Narges Gholami:** formal analysis. **Claudio Caria:** formal analysis, writing – review and editing. **Paola Bianchi:** formal analysis. **Cristiano Lauritano:** formal analysis. **Luigi Castello:** formal analysis. **Annalisa Roveta:** investigation, writing – review and editing. **Antonio Maconi:** investigation, writing – review and editing. **Stefano Andreoni:** investigation, conceptualization, project administration, writing – review and editing. **Sandra Dalfonso:** conceptualization, investigation, supervision, funding acquisition, project administration, resources, writing – review and editing. **Elisa Bona:** conceptualization, data curation, investigation, validation, formal analysis, visualization, writing – original draft, writing – review and editing.

## Ethics Statement

The study was approved by the Ethical Committee of Alessandria Center with protocol number 0009474 of 29/04/2022.

## Conflicts of Interest

The authors declare no conflicts of interest.

## Data Availability

The data that support the findings of this study are openly available in NCBI at https://submit.ncbi.nlm.nih.gov/subs/, reference number PRJNA1125274: SURVEIL Project.
